# In the Mouth of Madness: What Can a John Carpenter Horror Film Teach Us About Mental Health?

**DOI:** 10.1192/bjo.2026.11284

**Published:** 2026-06-30

**Authors:** Thomas Rourke

**Affiliations:** Black Country Healthcare NHS Foundation Trust, Wolverhampton, United Kingdom. Aston University, Birmingham, United Kingdom

## Abstract

**Aims:**

In the Mouth of Madness (1995) directed by John Carpenter, is a horror film in which through a nonlinear narrative and perspective of the protagonist, the writings of a horror author are discovered to cause madness and potentially physically change reality. This film is open to a variety of rich interpretations of the possible experience of mental illness and has been used by the researcher to teach medical students about mental illness and its psychopathology. The researcher aims to inspire other educators to utilise film and film analysis in their teaching as a useful and engaging tool that can aid with psychiatric teaching.

**Methods:**

Through interpretation of the five key elements of film, cinematography, Editing, mise-en-scène, performance and sound the film will be analysed. This analysis will be informed by the researcher's experience as a psychiatric trainee and of film analysis from completing a medical humanities degree.

**Results:**

This film shows the potential journey of a patient experiencing a first psychotic episode, providing informative examples of delusions of reference, passivity, nihilism and grandiosity, as well as a variety of perceptual disturbances. The film also provides an illustrative example of the societal stigma of mental illness and thoughtfully questions the viewer's assumptions, as exemplified in the following film quote:

*A reality is just what we tell each other it is. Sane and insane could easily switch places. If the insane were to become the majority you would find yourself locked up in a padded cell wondering what happened to the world.*

**Conclusion:**

This film analysis provides a rich source of material that can be used to educate and inform medical students about key aspects of psychopathology and lead them to question and examine their own and societal assumptions regarding mental illness. This analysis will hopefully inspire other educators to utilise film and film analysis in their teaching as a useful and engaging tool to aid with their psychiatric teaching.

